# Risk factors and prognosis for the development of acute kidney injury in patients using colistin in the intensive care unit: A retrospective cohort study

**DOI:** 10.1097/MD.0000000000036913

**Published:** 2024-01-12

**Authors:** Mustafa Deniz, Murat Alişik

**Affiliations:** a Intensive Care Unit, Izzet Baysal State Hospital, Bolu, Turkey; b Medical Biochemistry, Bolu Abant Izzet Baysal University, Bolu, Turkey.

**Keywords:** acute kidney injury, colistin, intensive care unit, multidrug resistance, vasopressors

## Abstract

Colistin, an antibiotic of polymyxin group, has recently been increasingly used in the treatment of multidrug resistant gram-negative bacteria. However, it has serious adverse effects such as acute kidney injury (AKI). We aimed to determine the factors affecting the development of AKI due to colistin, which has serious adverse effects, such as nephrotoxicity and neurotoxicity. We retrospectively analyzed the data of patients who received colistin for multidrug resistant gram-negative sepsis in adult intensive care units between January 2020 and December 2022. Demographic data, blood test results, concomitant drug use, need for renal replacement therapy, and mortality were recorded. Kidney damage was assessed according to the Kidney Disease Improving Global Outcomes criterion. We obtained data from 103 patients, 45 (43.7%) of whom were women. The most common comorbidity was a neurological disorder. Renal damage developed in 59.2% of patients. Renal replacement was required in 50.8% of the patients. Among patients who received colistin, 64.1% died. The use of vasopressors, diuretics, nephrotoxic agents with colistin, advanced age, and hypoalbuminemia were more common in patients with renal injury. Multivariate regression analysis showed that vasopressor use, prior creatinine elevation, and diuretic use were independent risk factors for colistin-induced AKI. Vasoactive agent use, previous kidney injury, and furosemide use were independent risk factors for colistin-induced nephrotoxicity. Considering these factors may be instructive for better monitoring of patients when colistin is required in intensive care units.

## 1. Introduction

Colistin, an antibiotic belonging to polymyxin group, is used in the treatment of multidrug-resistant (MDR) gram-negative microorganisms, mainly *Acinetobacter baumannii*.^[1]^ Although its use decreased due to its nephrotoxic and neurotoxic effects, the demand has increased again in recent years due to increasing MDR.^[2]^ Nephrotoxicity involves oxidative stress, inflammatory pathways, and apoptosis, and its incidence rate is up to 60% in patients using colistin.^[3]^ Reaching a consensus regarding the factors affecting kidney injury in patients receiving colistin is challenging due to the lack of clarity about the diseases, the use of different parameters in defining the kidney damage, and the use of multiple treatments and devices in intensive care units (ICUs).^[4,5]^ Sepsis is one of the most common causes of acute kidney injury (AKI) in ICUs, resulting in high morbidity and mortality. Further, polypharmacy, the use of vasoactive agents, and other effects of sepsis may increase AKI development and mortality.^[6,7]^

AKI is clinically defined according to the serum creatinine level and urine output. The Kidney Disease Improving Global Outcomes (KDIGO), an international consensus criterion, is the most commonly used definition. Risk, injury, failure, and end-stage (RIFLE) and Acute Kidney Injury Network (AKIN) criteria were used in defining AKI. Markers such as neutrophil gelatinase-associated lipocalin, IL-18, kidney injury molecule-1, and liver-type fatty acid-binding protein are indicators of tubular damage.^[8]^

This study aimed to define the development of AKI and factors affecting it based on the KDIGO criterion in patients receiving colistin in the ICUs.

## 2. Methods

We retrospectively analyzed the data of patients who received colistin for MDR gram-negative sepsis in adult ICUs of Bolu Izzet Baysal State Hospital between January 2020 and December 2022. Sex, comorbidities, age, Acute Physiology and Chronic Health Evaluation (APACHE II) score, Sequential Organ Failure Assessment (SOFA) score, blood tests performed on the day of colistin initiation (creatinine, urea, albumin, C-reactive protein (CRP), hemoglobin, platelets, white blood cells, and lymphocyte count), use of vasoactive or inotropic (noradrenaline) agents on the day of colistin initiation, antibiotics used before or together with colistin such as meropenem and vancomycin, use of furosemide, number of days for AKI development, renal replacement therapy (RRT), and mortality status were recorded by analyzing hospital automation systems and patient files. The KDIGO criterion was used to define AKI: Stage I: serum creatinine level 1.5 to 1.9 times or ≥0.3 mg/dL from baseline or the urine output <0.5 mL/kg for 6 to 12 hours; Stage II: serum creatinine level 2 to 2.9 times or the urine output 0.5 mL/kg for more than 12 hours; and Stage III: serum creatinine level 3-times the basal value or >4 mg/dL, RRT required, urine output <0.3 mL/kg for more than 24 hours, or anuria for more than 12 hours. Patients under 18 years of age, patients with COVID-19, patients with chronic renal failure, and those receiving colistin for <2 days were excluded from the study. The study was approved by the Bolu Abant Izzet Baysal University Clinical Research Ethics Committee (decision no. 2023/50, dated March 14, 2023).

### 2.1. Statistical analysis

Statistical analyses were performed using SPSS statistical software for Windows (version 20; IBM, Armonk, NY). The Kolmogorov–Smirnov test and distribution histograms were used to assess the normality of the assumption of distributions. The Mann–Whitney *U* test was used to compare non-normally distributed variables, and descriptive statistics are shown as median (1st–3rd quartile value). Pearson chi-squared test was used to compare categorical data. Spearman correlation test was used for the correlation analysis. The predictive values of these parameters were evaluated using multivariate regression analysis. Receiver operating characteristic (ROC) analysis was used to evaluate the classification ability of the parameters. Area under the curve (AUC) ± standard error, cutoff value, and sensitivity and specificity of the parameters were expressed using ROC analysis. *P* < .05 was considered statistically significant.

## 3. Results

We analyzed data of 1145 patients. Of these, 103 received colistin treatment, and 62 (59.2%) were diagnosed with AKI. Of 1042 patients who did not receive colistin treatment, 177 (17%) were diagnosed with AKI. Colistin was significantly found to cause AKI (*P* < .001). Of 103 patients, 45 (43.7%) were women who received colistin in the ICU. In terms of comorbidities, 35 (34%) patients had neurological disorders, including stroke, Alzheimer disease, and intracranial hemorrhage. Further, 28 (27.2%), 25 (24.3%), 24 (23.3%), and 22 (21.4%) patients had hypertension, chronic obstructive pulmonary disease, diabetes mellitus, and cardiac disease, respectively. The primary outcome of our study was the development of AKI, and 61 (59.2%) patients developed AKI. RRT was required in 31 (50.8%) patients. In our study, 66 (64.1%) patients died, which was a secondary outcome (Table 1).

**Table 1:** Demographics of study population.

	n	(%)
AKI	61	59.2
Mortality	66	64.1
Gender		
Female	45	43.7
Male	58	56.3
DM	24	23.3
HT	28	27.2
Cardiac disease	22	21.4
Neurologic disorders	35	34
COPD	25	24.3
RRT (n = 61)	31	50.8
Pneumonia	49	47.6
Blood stream infection	32	31
Urinary tract Infection	12	11.6
Pressure ulcer infection	10	9.7
*Acinetobacter* spp	38	36.9
*Pseudomonas* spp	14	13.6
*Klebsiella* spp	30	29.1
None	21	20.3

AKI = acute kidney injury, COPD = chronic obstructive pulmonary disease, DM = diabetes mellitus, HT = hypertension, RRT = renal replacement therapy.

Compared with the colistin-treated patients without AKI (AKI(−) group), age (*P* = .009), APACHE II score (*P* < .001), and SOFA score (*P* < .001) were significantly higher in the colistin-treated patients with AKI (AKI(+) group). Further, serum creatinine (*P* < .001), urea (*P* < .001), and CRP (*P* < .001) were significantly higher in the AKI(+) group than the AKI(−) group, whereas serum albumin (*P* < .001), hemoglobin (*P* < .001), and platelet (*P* = .001) levels were significantly lower in the AKI(+) group. In addition, mortality was higher in the AKI(+) group (*P* < .001), and no significant differences were observed between the groups in terms of sex and comorbidities. In the AKI(+) group, the number of patients who received a noradrenaline infusion (*P* < .001), meropenem (*P* = .001), and furosemide (*P* = .016) on the day of colistin initiation was significantly higher (Table 2).

**Table 2:** Comorbidity, blood tests, and drug usage of patients with and without AKI.

	AKI(−)	AKI(+)	*P* value
Age (yr)	66 (55–79.3)	77 (65.5–85.5)	.009
APACHE II	16 (15–20.8)	22 (20–24)	<.001
SOFAs	7 (6–9.3)	9 (8–10.5)	<.001
Creatinine (mg/dL)	0.8 (0.6–1)	1.4 (1–2.1)	<.001
Urea (mg/dL)	40.5 (32.8–59.3)	70 (48–127.5)	<.001
Albumin (g/L)	25.5 (21.8–30.3)	20 (18–23)	<.001
CRP (mg/L)	87 (49.5–113.5)	120 (106.5–126)	<.001
Hgb (K/μL)	9.9 (9.4–12)	8.3 (7.8–9)	<.001
Platelets (K/μL)	252 (193.3–337.8)	185 (109–259.5)	.001
WBC (K/μL)	10 (8.8–14)	12 (9.4–14)	.197
Lymphocyte (K/μL)	1.1 (0.9–1.3)	1.1 (0.6–1.6)	.628
Mortality	12 (28.6%)	54 (88.5%)	<.001
Gender			
Female	16 (38.1%)	29 (47.5%)	.342
Male	26 (61.9%)	32 (52.5%)	
DM	8 (19%)	16 (26.2%)	.397
HT	9 (21.4%)	19 (31.1%)	.276
Cardiac disease	6 (14.3%)	16 (26.2%)	.146
Neurologic disorders	14 (33.3%)	21 (34.4%)	.908
COPD	10 (23.8%)	15 (24.6%)	.928
Noradrenaline	10 (23.8%)	53 (86.9%)	<.001
Meropenem	20 (47.6%)	49 (80.3%)	.001
Vancomycin	6 (14.3%)	14 (23%)	.275
Furosemide	12 (28.6%)	32 (52.5%)	.016

AKI = acute kidney injury, APACHE = Acute Physiology and Chronic Health Evaluation, COPD = chronic obstructive pulmonary disease, CRP = C-reactive protein, DM = diabetes mellitus, Hgb = hemoglobin, HT = hypertension, SOFAs = Sequential Organ Failure Assessment score, WBC = white blood cell.

When we analyzed the 2 groups as survival and deceased, no significant difference was found between the 2 groups in terms of sex and comorbidities. AKI (*P* < .001), noradrenaline use on the day of colistin initiation (*P* < .001), meropenem use on the day of colistin initiation (*P* = .011), age (*P* < .001), APACHE II score (*P* < .001), SOFA score (*P* < .001), serum CRP level (*P* < .001) and serum urea level (*P* = .004) were significantly higher in the deceased group than the survival group. Further, serum lymphocyte count (*P* = .03) and serum albumin level (*P* < .001) were significantly lower in the deceased group. Patients who developed AKI and required RRT (*P* < .001) and patients who developed AKI after colistin use (*P* = .039) were significantly higher in the deceased group (Table 3).

**Table 3:** Comorbidity, blood tests, APACHE II, SOFAs, AKI status, and drug usage of patients deceased and survived groups.

	Survived	Deceased	*P* value
AKI	7 (16.7%)	54 (88.5%)	<.001
Gender			
Female	14 (33.3%)	31 (50.8%)	.37
Male	23 (54.8%)	35 (57.4%)	
DM	8 (19%)	16 (26.2%)	.763
HT	8 (19%)	20 (32.8%)	.342
Cardiac disease	6 (14.3%)	16 (26.2%)	.34
Neurologic disorders	13 (31%)	22 (36.1%)	.853
COPD	7 (16.7%)	18 (29.5%)	.343
Noradrenaline	5 (11.9%)	58 (95.1%)	<.001
Meropenem	19 (45.2%)	50 (82%)	.011
Vancomycin	4 (9.5%)	16 (26.2%)	.123
RRT	3 (7.1%)	28 (45.9%)	<.001
AKI (d)	5 (4–8)	4 (3–4)	.039
Age (yr)	59 (54–70)	79 (67.8–86)	<.001
APACHE II	16 (15–19.5)	22 (21–25)	<.001
SOFAs	7 (5–8)	9.5 (9–11)	<.001
Creatinine (mg/dL)	0.9 (0.6–1.5)	1.1 (0.8–1.7)	.114
Urea (mg/dL)	41 (33.5–90.5)	68.5 (45.8–123.3)	.004
Albumin (g/L)	26 (22.5–31.5)	20 (18–23)	<.001
CRP (mg/L)	88 (69.5–111)	121 (101–128)	<.001
WBC (K/μL)	11 (8.5–14)	11.3 (9.3–14)	.563
Lymphocyte (K/μL)	1.1 (1–2.2)	1.0 (0.6–1.4)	.03

AKI = acute kidney injury, APACHE = Acute Physiology and Chronic Health Evaluation, COPD = chronic obstructive pulmonary disease, CRP = C-reactive protein, DM = diabetes mellitus, HT = hypertension, RRT = renal replacement therapy, SOFAs = Sequential Organ Failure Assessment score, WBC = white blood cell.

A multivariate logistic regression analysis revealed that noradrenaline infusion on the day of colistin initiation (*P* = .003), high serum creatinine level (*P* = .003), and furosemide use (*P* = .032) were the risk factors for the development of AKI in patients administered colistin (Table 4). Further ROC analysis of these parameters revealed that a serum creatinine cutoff value of >1.1 was associated with a sensitivity of 70.49%, specificity of 83.33%, and AUC of 0.809 (95% CI); serum albumin cutoff value of <25 was associated with a sensitivity of 59.52%, specificity of 91.8%, and AUC of 0.807 (95% CI); serum CRP cutoff value of >91 was associated with a sensitivity of 90.16%, specificity of 61.9%, and AUC of 0.746 (95% CI); and serum urea cutoff value of >45 was associated with a sensitivity of 86.89%, specificity of 66.67%, and AUC of 0.781 (95% CI) (Table 5).

**Table 4:** Multivariate logistic regression analysis of parameters.

Parameters	*B* (SE)	Wald	Sig.	OR (95% CI)
AGE (yrs)	−0.012 ± 0.043	0.077	0.781	0.988 (0.907–1.076)
APACHE II	−0.123 ± 0.165	0.551	0.458	0.885 (0.64–1.223)
SOFAs	0.843 ± 0.547	2.378	0.123	2.324 (0.796–6.784)
Creatinine (mg/dL)	7.951 ± 2.701	8.665	0.003	2838.803 (14.253–565,421.155)
CRP (mg/L)	0.009 ± 0.022	0.165	0.685	1.009 (0.966–1.054)
Albumin (g/L)	0.192 ± 0.173	1.231	0.267	1.211 (0.863–1.7)
Lymphocyte (K/μL)	2.239 ± 1.391	2.590	0.108	9.381 (0.614–143.312)
Noradrenaline	10.067 ± 3.407	8.728	0.003	23,550.833 (29.619–18,725,719.677)
Furosemide	5.74 ± 2.67	4.623	0.032	311.018 (1.661–58,228.407)

Nagelkerke *R*^2^ = 0.878.

APACHE = Acute Physiology and Chronic Health Evaluation, *B* = beta, CI = confidence interval, CRP = C-reactive protein, OR = odd ratio, SE = standard error, SOFAs = Sequential Organ Failure Assessment score.

**Table 5:** ROC analysis of parameters.

Parameters	Cutoff	AUC (95% CI)	Sensitivity (%)	Specificity (%)	*P* value
APACHE II	>21	0.755 (0.650–0.861)	73.77	76.19	<.001
SOFAs	>9	0.747 (0.642–0.853)	73.77	73.81	<.001
Creatinine (mg/dL)	>1.1	0.809 (0.725–0.893)	70.49	83.33	<.001
Urea (mg/dL)	>45	0.781 (0.685–0.876)	86.89	66.67	<.001
Albumin (g/L)	<25	0.807 (0.718–0.896)	59.52	91.8	<.001
CRP (mg/L)	>91	0.746 (0.635–0.857)	90.16	61.9	<.001
Hgb (K/μL)	<9.2	0.823 (0.738–0.907)	80.95	80.33	<.001
Platelets (K/μL)	<201	0.700 (0.598–0.802)	71.43	67.21	.001
WBC (K/μL)	>10.3	0.575 (0.457–0.693)	70.49	52.38	.198
Lymphocyte (K/μL)	<1.0	0.528 (0.416–0.640)	73.81	47.54	.629

APACHE = Acute Physiology and Chronic Health Evaluation, AUC = area under the curve, CI = confidence interval, CRP = C-reactive protein, Hgb = hemoglobin, SOFAs = Sequential Organ Failure Assessment score, WBC = white blood cell.

## 4. Discussion

Colistin, which was withdrawn from use due to its serious adverse effects, has begun to be reused owing to the increasing carbapenem resistance of microorganisms and MDR. During prolonged hospitalizations in ICUs, gram-negative microorganisms, such as *A baumannii*, cause conditions such as pneumonia, sepsis, and septic shock, which may result in mortality. AKI can be influenced by drugs, conditions related to the patient in the ICU, and comorbidities.

Shields et al^[9]^ investigated the development and incidence of AKI in patients treated with colistin and found that 12% and 29% of patients developed AKI on day 2 and 7, respectively. Further, they observed that chronic liver disease, vancomycin use with colistin, and high-dose colistin were risk factors, and the KDIGO criterion was useful for the early diagnosis of AKI. Arrayasillapatorn et al^[1]^ retrospectively analyzed the data of 412 patients and found that AKI developed in 68.5% of patients and that advanced age, the presence of previous AKI or elevated creatinine levels, and the use of vasopressors, diuretics, and vancomycin when colistin was initiated were the risk factors. In their study, colistin-induced AKI was associated with mortality.^[1]^ Ozel et al^[10]^ applied colistin loading and maintenance therapy in their observational study and investigated colistin-associated renal damage using the RIFLE criterion. They found that preexisting high creatinine levels, advanced age, and high plasma colistin levels were risk factors for nephrotoxicity.

Khalifeh et al^[11]^ diagnosed colistin-related AKI in 46.9% of 113 patients using the KDIGO criterion and found that low serum albumin levels and the simultaneous use of 2 or more nephrotoxic agents with colistin were risk factors for AKI. Al-Abdulkarim et al^[12]^ defined renal damage according to the RIFLE criterion in patients receiving colistin for more than 72 hours. Colistin-related nephrotoxicity developed in 45.7% of the 70 patients. They identified advanced age and high-dose colistin use as the risk factors. In a laboratory study, intraperitoneal colistin was administered to mice, and increased levels of blood urea nitrogen, renal damage markers, and cell apoptosis were observed. In the same mouse group, the 200 µg/mL colistin dose significantly increased cell apoptosis.^[13]^ Rabi et al^[14]^ investigated the risk factors and the time of AKI development and found that patients who were initiated with colistin due to *A baumannii*, patients who used furosemide before and concomitantly, older patients, overweight patients, and patients with hypoalbuminemia were at higher risk for AKI development. In the same study, the mortality rate was higher in patients who developed colistin-induced AKI.

Kucuk et al^[15]^analyzed the relationship between dexmedetomidine and AKI in patients receiving colistin and found that 47% of the patients developed AKI after a mean of 5 days. In this study, advanced age, low glomerular filtration rate, and vasopressor use were associated with AKI development, whereas dexmedetomidine use was independently associated with lower AKI development. Notably, colistin was found to be unrelated to both AKI development and mortality when AKI was analyzed using the AKIN in patients receiving colistin and combination antibiotic therapy for MDR ventilator-associated pneumonia.^[16]^ Peng et al^[17]^ examined adverse effects and mortality in patients receiving colistin in a multicenter study of 122 patients. They found that high SOFA scores, prolonged hospitalization, and the need for extracorporeal membrane oxygenation were independent risk factors for mortality, with a mortality rate of 29.5%. In the same study, AKI developed in 5 patients with previously high creatinine levels, according to KDIGO. In a multicenter study, Korkmaz Erken et al^[18]^ investigated AKI using the RIFLE criterion in patients receiving colistin and found that 62.3% of patients developed AKI, with 66.9% mortality. In their study, the risk factors for AKI were high-dose colistin use, advanced age, previous AKI, hypertension, and male sex.

Rivero et al^[19]^ evaluated renal damage in COVID-19 patients analyzing renal biopsy results and observed tubular epithelial loss due to nephrotoxic agents such as colistin using electron microscopy. Mazzitelli et al^[20]^ compared cefiderocol-based versus colistin-based treatment regimens in their study, referring to the increase in carbapenem resistance during the COVID-19 pandemic, and found that AKI development was higher in patients using colistin between the 2 groups; however, no difference was observed in mortality. In a review, Topete et al^[21]^ stated that COVID-19 resulted in the increase the number of critically ill patients and evaluated the adverse effects and drug resistance inpatients using colistin. They concluded that critical patients with COVID-19 could be added to conditions such as hypoalbuminemia, septic shock, age, etc, which are effective in colistin nephrotoxicity.

In our study, we analyzed the data of 103 patients treated with colistin for gram-negative MDR microorganisms. The AKIN, RIFLE, and the current version of the KDIGO criteria have been used in several studies. In our study, we used KDIGO as the AKI criterion. According to the KDIGO, AKI developed in 59.2% of patients, and RRT was required in 50.8% of these patients. The fact that almost 1 of every 2 patients did not require RRT suggests that these patients had stage-I–II AKI. In our study, the mortality rate was high (64.1%) in patients who developed colistin-induced AKI. Compared with the patients who did not develop AKI, we found that patients who developed AKI were older, had higher APACHE II and SOFA scores, had previously impaired renal function, had low serum albumin levels, and received nephrotoxic agents such as vasoactive agents, diuretics, and meropenem on the day of colistin initiation. A multivariate logistic regression analysis revealed that noradrenaline infusion, furosemide, and high serum creatinine levels on the day of colistin initiation were independent risk factors for AKI development in patients receiving colistin. Vasoactive agents increase blood pressure and impair tissue perfusion through vasoconstriction, which may increase the damaging capacity of colistin, an agent that promotes apoptosis. We believe that patients with renal damage due to other causes and impaired serum renal function may be more sensitive to colistin nephrotoxicity. Further, furosemide may increase the sensitivity to colistin because it may dysregulate the intravascular volume and cause electrolyte disturbance and AKI in critically ill patients. In our study, sex and comorbidities were not associated with AKI development.

### 4.1. Conclusion

Colistin is an antibiotic that has recently been increasingly used in the treatment of MDR gram-negative bacteria; however, it has serious adverse effects such as AKI. In this study, we analyzed the effective conditions for the development of AKI in patients receiving colistin. We found that patients with older age and hypoalbuminemia were at risk for AKI. Using multivariate regression analysis, we found that the use of vasoactive agents, elevated creatinine levels, and furosemide use were independent risk factors for AKI development. Notably, more randomized controlled studies are needed to define the risk factors for AKI.

### 4.2. Limitations

Our study has some limitations. First, it was a retrospective study. Second, we could not evaluate the intravascular volume status associated with renal injury. Third, although we determined the number of vasoactive agents used, we could not specify the infusion dose per kilogram. Further, we could not specify the colistin plasma level or colistin dose per kilogram. In addition, we could not record the potential nephrotoxic agents used by patients with comorbidities for a long time before ICU admission. Fourth, we did not specify the AKI stages according to the KDIGO criterion.

## Author contributions

**Conceptualization:** Mustafa Deniz, Murat Alişik.

**Data curation:** Mustafa Deniz, Murat Alişik.

**Formal analysis:** Mustafa Deniz, Murat Alişik.

**Funding acquisition:** Mustafa Deniz, Murat Alişik.

**Investigation:** Mustafa Deniz, Murat Alişik.

**Methodology:** Mustafa Deniz, Murat Alişik.

**Project administration:** Mustafa Deniz, Murat Alişik.

**Resources:** Mustafa Deniz.

**Software:** Mustafa Deniz, Murat Alişik.

**Supervision:** Mustafa Deniz, Murat Alişik.

**Validation:** Mustafa Deniz.

**Visualization:** Mustafa Deniz, Murat Alişik.

**Writing – original draft:** Mustafa Deniz, Murat Alişik.

**Writing – review & editing:** Mustafa Deniz.
